# Simultaneous inhibition of aryl hydrocarbon receptor (AhR) and Src abolishes androgen receptor signaling

**DOI:** 10.1371/journal.pone.0179844

**Published:** 2017-07-03

**Authors:** Maryam Ghotbaddini, Keyana Cisse, Alexis Carey, Joann B. Powell

**Affiliations:** Clark Atlanta University-Center for Cancer Research and Therapeutic Development, Atlanta, Georgia, United States of America; Seconda Universita degli Studi di Napoli, ITALY

## Abstract

Altered c-Src activity has been strongly implicated in the development, growth, progression, and metastasis of human cancers including prostate cancer. Src is known to regulate several biological functions of tumor cells, including proliferation. There are several Src inhibitors under evaluation for clinical effectiveness but have shown little activity in monotherapy trials of solid tumors. Combination studies are being explored by in vitro analysis and in clinical trials. Here we investigate the effect of simultaneous inhibition of the aryl hydrocarbon receptor (AhR) and Src on androgen receptor (AR) signaling in prostate cancer cells. AhR has also been reported to interact with the Src signaling pathway during prostate development. c-Src protein kinase is associated with the AhR complex in the cytosol and upon ligand binding to AhR, c-Src is activated and released from the complex. AhR has also been shown to regulate AR signaling which remains functionally important in the development and progression of prostate cancer. We provide evidence that co-inhibition of AhR and Src abolish AR activity. Evaluation of total protein and cellular fractions revealed decreased pAR expression and AR nuclear localization. Assays utilizing an androgen responsive element (ARE) and qRT-PCR analysis of AR genes revealed decreased AR promoter activity and transcriptional activity in the presence of both AhR and Src inhibitors. Furthermore, co-inhibition of AhR and Src reduced the growth of prostate cancer cells compared to individual treatments. Several studies have revealed that AhR and Src individually inhibit cellular proliferation. However, this study is the first to suggest simultaneous inhibition of AhR and Src to inhibit AR signaling and prostate cancer cell growth.

## Introduction

The Src-family tyrosine kinases (SFKs) are oncogenic enzymes that contribute to the initiation and progression of many types of cancer, including prostate cancer. Src plays an important role in cell proliferation, differentiation, adhesion, and migration. Src has been identified as a potent and specific therapeutic target for prostate cancer progression [1].

Other than skin cancer, prostate cancer is the most common cancer in American men. An estimated 1 in 6 men will be diagnosed with prostate cancer and 1 in 36 will die from the disease. Such rates establish prostate cancer as the second leading cause of all cancer-related deaths in men [2]. During progression to an androgen-independent state, prostate cancer cells continue to express the androgen receptor (AR) and androgen-regulated genes, indicating that AR is critical for the proliferation of castration-resistant prostate cancer (CRPC) cells [3]. CPRC is defined by rising prostate-specific antigen (PSA) levels or progressive disease in the presence of castrate testosterone levels. CRPC appears to continue to rely on the AR for growth and progression [4].

AR is a member of the steroid hormone receptor family which is primarily responsible for mediating the physiological effects of androgens by binding to specific DNA sequences, known as androgen response elements (AREs) [5]. The AR protein has multiple phosphorylation sites that regulate nuclear localization [6,7,8]. Several studies have shown the Src mediated phosphorylation of AR resulting in transcriptional activation of AR in the absence of androgens. Particularly, AR was transcriptionally activated by Src-mediated phosphorylation of AR at Y534 in the absence of androgen [9,10,11].

Moreover, Src has also been reported to interact with other pathways such as the aryl hydrocarbon receptor (AhR) signaling pathway during prostate development [12]. c-Src protein kinase is associated specifically with the AhR complex along with heat shock protein 90 (hsp90) in the cytosol and following ligand binding to the Ah-receptor subunit, c-Src is activated and released from the complex [12]. AhR is constitutively active in advanced prostate cancer cell lines that model CRPC and where Src activity is also elevated. AhR helps to sustain androgen-independent growth of prostate cancer cells. Attenuation of AhR activity reduces expression of phosphorylated AR, androgen responsive genes and androgen mediated growth. Rapid activation of c-Src kinase following treatment with an AhR ligand has been reported in several different cell lines and may be required for AhR mediated regulation of AR activity [13].

Because Src is highly expressed in the majority of PCa specimens, Src inhibitors are an attractive therapeutic target for men with metastatic PCa [14]. However, Src inhibitors have shown little activity in monotherapy trials and combination studies are being conducted to further evaluate the effect of Src inhibition in solid tumors. The importance of c-Src kinase activity for aryl hydrocarbon receptor (AhR) signaling has been demonstrated and may identify AhR as a target in combination therapy. Considering the level of crosstalk that occurs between AhR, Src and AR, co-targeting AhR and Src may be an effective strategy to abolish uncontrolled AR activity in CRPC.

## Materials and methods

### Chemical and reagents

AhR antagonist, (CH223191) was purchased from Sigma Aldrich. Src kinase inhibitor, protein phosphatase 2 (PP2) was purchased from Sigma Aldrich.

### Cell culture

Adherent monolayer cultures of C4-2 human prostate cancer cell lines (Dr. Valerie Odero-Marah, Clark Atlanta University, Atlanta, GA) were maintained in RPMI 1640 medium supplemented with 10% FBS. Cells were grown at 37°C with 5% CO2 in humidified atmosphere, and media was replaced every other day. Cells were split (1:3), when they reached near confluence.

### Protein isolation and western blot analysis

24 hours after treatment with AhR and Src inhibitors, protein samples were isolated using the Thermo Scientific NE-PER Extraction kit for cellular fractions or commercially available cell lysis buffer (Cell Signaling) for total protein. Total protein was isolated from (C4-2) SCR and–AhR cells at 75% confluency without treatment. Protein samples were resolved by SDS-PAGE and transferred to a PVDF membrane. Immunoblotting was carried out with 200 μg/ml mouse AhR monoclonal antibody (Santa Cruz) at 1:500 dilution in 5% milk, 200 μg/ml mouse AR monoclonal antibody (Santa Cruz) at 1:50 dilution in 5% milk, 100 μg/ml mouse pAR monoclonal antibody (GenScript-Piscataway, NJ) at 1:50 dilution in 5% milk, and 100 μg/ml rabbit pSrc monoclonal antibody (Santa Cruz) at 1:1000 dilution in 5% BSA. Blots were washed three times (10 min each) with TBST. The blots were then incubated in 1:2500 dilution of secondary antibody and washed three times (15 min each) with TBS. Bands were visualized with the enhanced chemiluminescence (ECL) kit as specified by the manufacturer. Multiple exposures of each set of samples were produced. The relative concentration of target protein was determined by computer analysis using image J and normalized to an internal standard (topoisomerase, β-tubulin, β-actin).

### RNA extraction and quantitative qRT-PCR analysis

Total RNA was isolated from cell monolayers grown in 100 mm tissue culture dishes using RNeasy Mini Kit (Qiagen) 24 hours after cells were treated with AhR and Src inhibitors. 2 μg of the total RNA was reverse-transcribed using the Superscript II kit (Invitrogen), according to the manufacturer’s recommendations. The cDNA served as a template in a 25 μl reaction mixture and was processed using the following protocol: an initial denaturation at 95°C for 3 min, followed by 39 amplification cycles (95°C for 10 s and 55–65°C for 30 s), 95°C for 10 s, 65°C for 5 s and 95°C for 50 s. The 25 μl qPCR reaction mixture was mixed with GoTaq qPCR Master Mix (Promega). Melt curve analyses were performed after each run to ensure a single product. Relative gene expression was determined using the ΔΔCq calculation method. The primer sequences used were: CYP1B1: Forward (5′-3′) TGCCTGTCACTATTCCTCATGCCA & Reverse (5′-3′) TCTGCTGGTCAGGTCCTTGTTGAT. AhR Forward (5′-3′) TCCTTGGCTCTGAACTCAAGCTGT & Reverse (5′-3′) GCTGTGGACAATTGAAAGGCACGA. KLK3 Forward (5′-3′) ACTTCAGTGTGTGGACCTCCATGT & Reverse (5′-3′) AGCACACAGCATGAACTTGGTCAC. AR: Forward (5′-3′) GAGCTAGCCGCTCCAGTGCT & Reverse (5′-3′) CCTAACCAGGCGGGTCGTGG. Primers to amplify the 470-bp cDNA fragment encoding L19 were used as an internal control. L-19: Forward (5′-3′) TCCCAGGTTCAAGCGATTCTCCTT & Reverse (5′-3′) TTGAGACCAGCCTGACCAACATGA.

### Proliferation studies

Growth of cells was assayed using the Promega CellTiter 96 Cell Proliferation Assay. Cells were resuspended to a final concentration of 1.0 x 10^5^/mL in RPMI. 50 μl of the cell suspension (5,000 cells) was added to each well of the 96-well plate containing 50 μl of media with corresponding treatment resulting in a total volume of 100 μl. The micro plates were incubated at 37°C for 24–72 hours in a humidified, 5% CO2 atmosphere. Per manufacturer’s instructions, following incubation, 20 μl of MTS / PMS solution was added to each well and incubated for 4 hours. Absorbances were read at 490 nm using the Synergy H1m multimode micro plate reader.

### Cell cycle analysis

Cells were trypsinized and single cell suspensions were fixed in cold 100% ethanol for 1hour at 4°C. Cells were washed and collected by centrifugation at 700rpm for 5 minutes and resuspended in PBS. The cells were checked microscopically to ensure no clumps persisted. If clumps were observed, cells were passed 3–5 times through a 25-gauge syringe needle. RNase in PBS (0.01 mg/ml) and propidium iodide (40 μg/ml) was added to the suspension. The cells were incubated at 4°C for 3 hours. The fluorescent cells were then analyzed in an Accuri C6 Flow Cytometer (Becton-Dickson). The percentage of cell cycle distribution was determined using FlowJo analysis software.

### XRE and ARE promoter activity

4 x 10^4^ Cells were plated in a 96 well plate. C4-2 cells were transfected with XRE and ARE reporter, as well as with positive and negative control reporter plasmids using attractene. After 16 hours of transfection, media was changed to standard assay media (DMEM +0.5% FBS +0.1 mM NEAA). Cells were grown for an additional 8 hours under normal cell conditions. After 24 hours of transfection cells were treated with AhR and Src inhibitors and then harvested 18 hours after treatment. A dual luciferase assay was performed after 42 hours of transfection, and promoter activity values are expressed as arbitrary florescence units (AFU). Experiments were performed in triplicate and the standard error is indicated.

### Statistical analysis

Each experiment was carried out in triplicate and all the values are expressed as mean +SEM. The differences between the groups were compared by t-test or ANOVA using Instant software (GraphPad Software Inc., San Diego, CA). A value of P<0.05 was considered statistically significant.

## Results

### Co-inhibition of AhR and Src abolishes phosphorylation of AR

**Fig 1:**
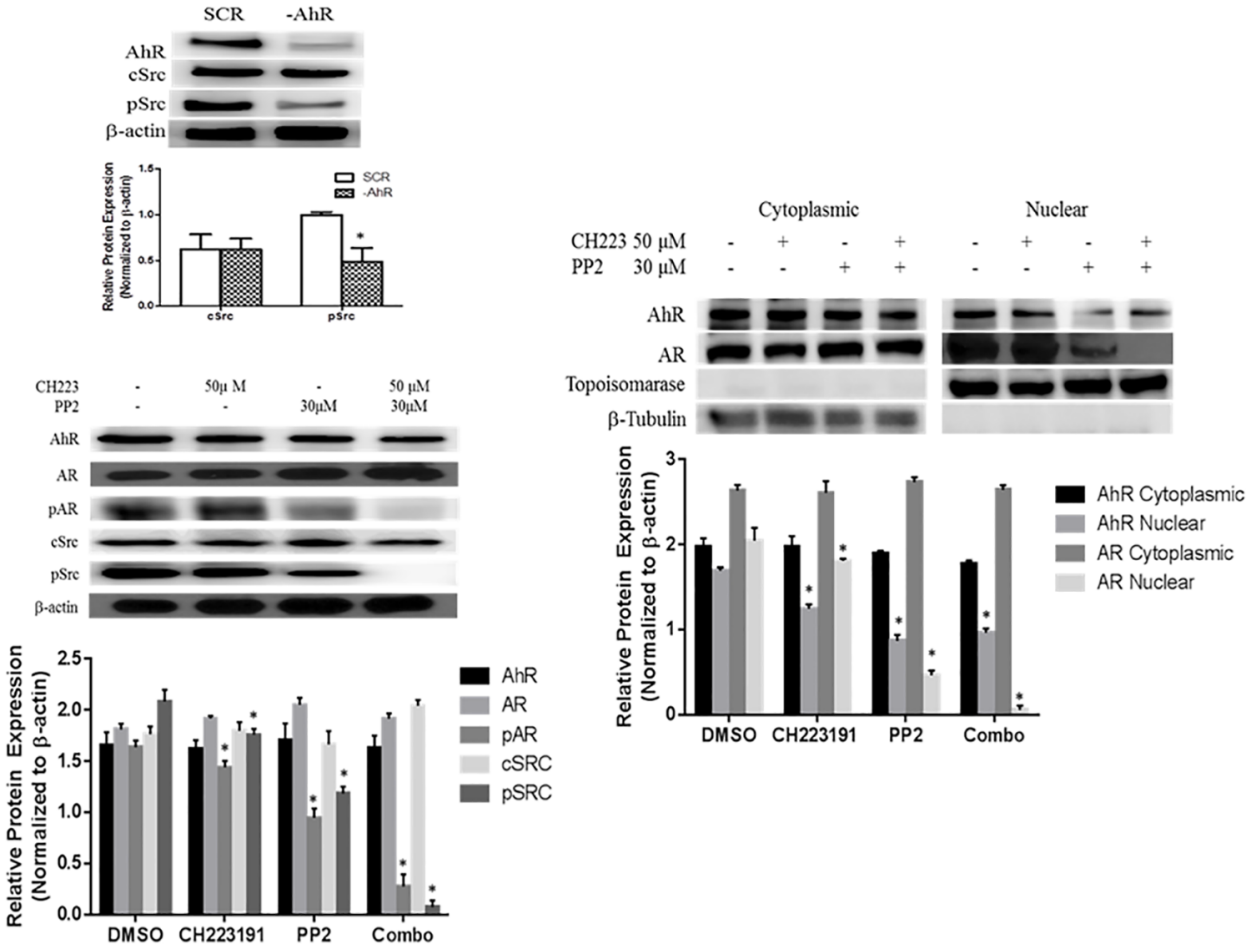
AhR and Src inhibition synergistically reduces pAR expression and nuclear localization. (A) C4-2 cells were transfected with a vector carrying a shRNA for AhR (-AhR) or a scrambled control vector (SCR). Total cellular proteins were isolated from 75% confluent cell cultures and separated by SDS polyacrylamine gel electrophoresis and blotted using anti-AhR antibody, anti-cSrc antibody, anti-pSrc antibody. Anti-β-actin was used as a loading control. Parental C4-2 prostate cancer cells were also treated with AhR inhibitor (CH223191) and Src kinase inhibitor (PP2) alone and in combination. DMSO served as a vehicle control. (B) Total cellular proteins were isolated and proteins were separated by SDS polyacrylamine gel electrophoresis and blotted using anti-AhR antibody, anti-AR antibody, anti-pAR antibody, anti-cSrc antibody, anti-pSrc antibody. Anti-β-actin was used as a loading control. Figure is representative of 3 independent membranes. (C) Nuclear and cytoplasmic fractionation: C4-2 cells grown on 100 mm dishes until ∼75% confluent were treated with DMSO, CH223191 and PP2 as described above. Cells were washed with cold PBS and cellular fractions were isolated per manufactures instructions using a NE-PER Extraction kit. The nuclear and cytoplasmic fractions were analyzed by western blotting for AhR and AR protein expression. The relative level of cytoplasmic AhR & AR were normalized with β-tubulin expression and the relative level of nuclear AhR AR were normalized with topoisomerase expression. Blots are representative of three independent experiments.

C4-2 prostate cancer cells were used as a CRPC cell model [15]. These cells were isolated from a chimeric tumor induced by inoculating a castrated mouse with parental androgen sensitive LNCaP cells [16]. AhR was previously reported to be constitutively active in C4-2 cells and activation of Src kinase has been verified to accompany AhR activity. We previously reported decreased pAR in C4-2 cells with shRNA mediated reduction in AhR protein expression. Here, western blot analysis reveals a corresponding decrease in pSrc expression (Fig 1A) [13, 15]. In order to determine the effects of chemical inhibition of AhR and Src C4-2 were treated with 4-amino-5-(4-chlorophenyl)-7-(dimethylethyl) pyrazolo[3,4d]pyrimidine (PP2) and specific AhR inhibitor (CH223191). PP2 is a widely used compound to block the activity of Src family kinases, reduced phosphorylation of Src and AR in C4-2 cells [17]. The addition of specific AhR antagonist CH223191 abolished both Src and AR phosphorylation in the presence of PP2. The expression levels of non-phosphorylated AR and Src were not affected with treatment. [Fig 1B].

### Nuclear AR is AhR / Src dependent

The translocation of AR into the nucleus is dependent upon the phosphorylation of AR. In order to address the role of AhR and Src in this process cellular fractions were analyzed for AhR expression by western blot to determine sub-cellular localization. Individually, PP2 and CH223191 reduced the presence of AR in the nucleus with a greater effect seen with PP2. However, simultaneous inhibition of AhR and Src abolished AR nuclear localization [Fig 1C].

### Co-inhibition of AhR and Src decreases promoter activity of AhR and AR

**Fig 2:**
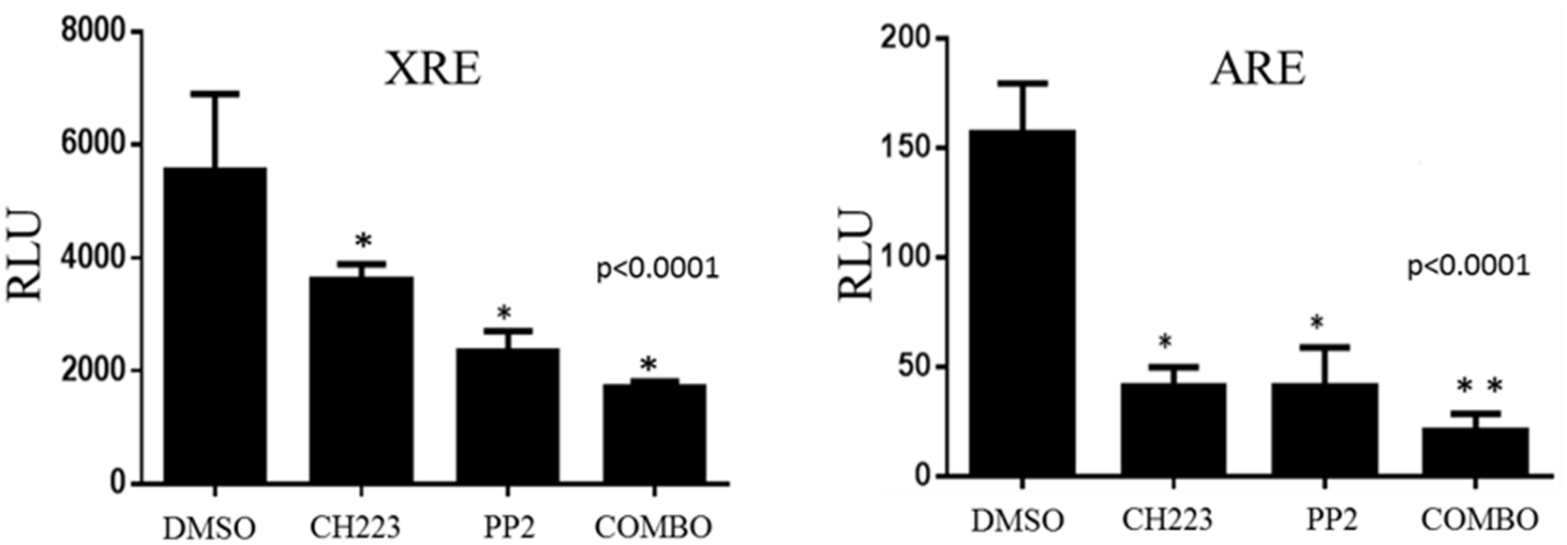
Simultaneous inhibition of AhR and Src significantly decrease AR and AhR promoter activity. C4-2 cells were transfected with an XRE reporter plasmid & ARE reporter plasmid, as well as with positive and negative control reporter plasmids using attractene. Following transfection, treatments were added to each appropriate plate. A dual luciferase assay was performed and promoter activity values are expressed as arbitrary florescence units (AFU). Each bar represents mean ± SEM (n  =  3) and were analyzed by student t-test. (*) denotes statistically significant differences (*P<0.0001).

The Cignal XRE and ARE luciferase reporter assays were utilized to determine the activity of AhR and AR signaling pathways in androgen independent (C4-2) prostate cancer cells in the presence and absence of inhibitors. The assay showed that C4-2 prostate cancer cells have a high level of AhR and AR promoter activity in the absence of inhibitor treatment. CH223191 reduced AhR promoter activity by 30% while PP2 resulted in a 50% decrease in promoter activity compared to DMSO. Both CH223191 and PP2 reduced AR promoter activity by 70% compared to the DMSO treated cells. Simultaneous inhibition of AhR and Src in C4-2 cells with CH223191/PP2 resulted in minimal AR promoter activity. Co-treatment with CH223191 and PP2 significantly decreased AR promoter activity compared to CH223191 or PP2 alone [Fig 2].

### Synergistic inhibition of AhR and AR target gene expression

**Fig 3:**
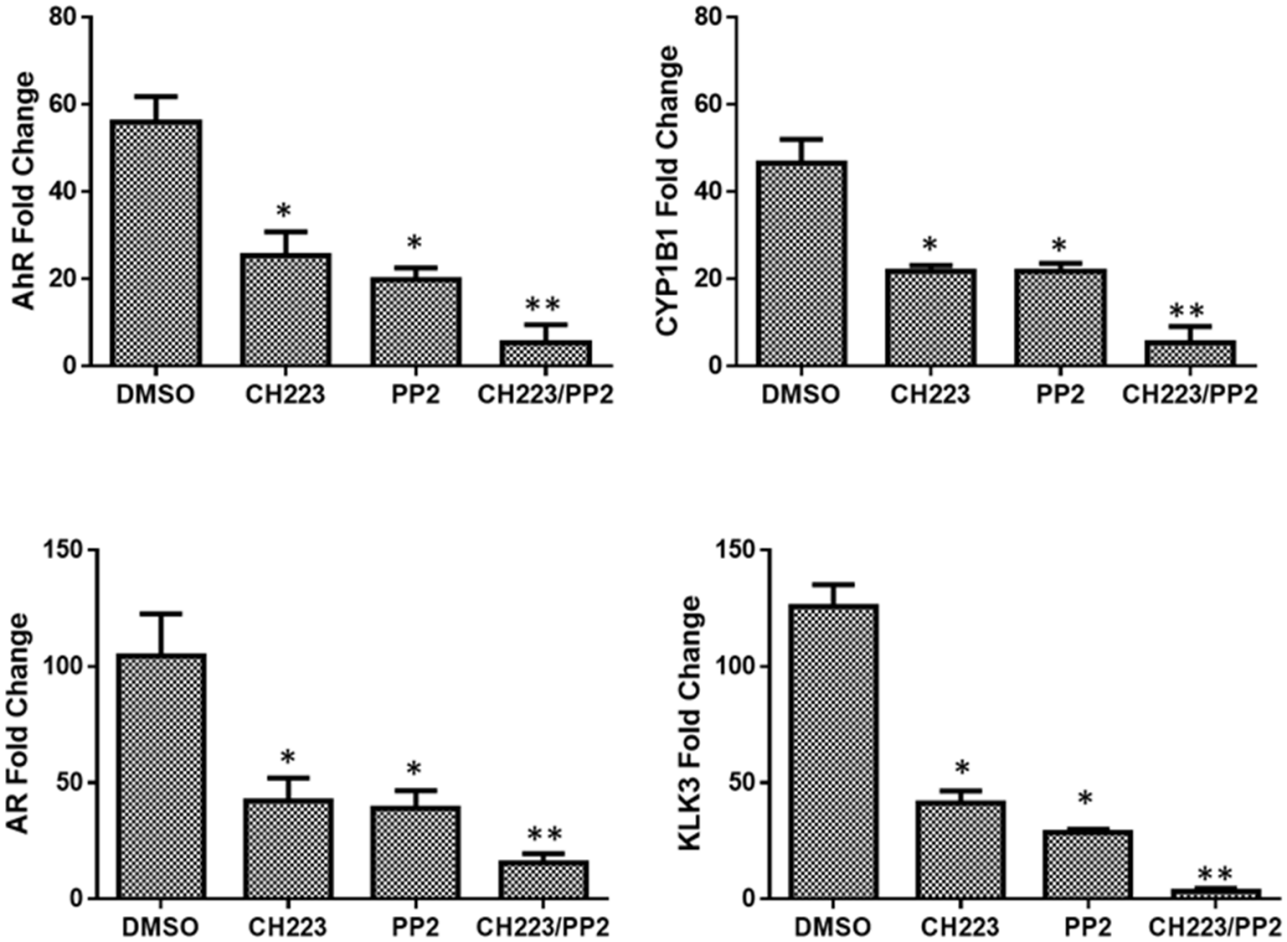
AhR and AR transcriptional activity synergistically ablated by CH223191 and PP2. qRT-PCR analysis examined AhR, CYP1B1, CYP1A1, AR, KLK2 and KLK3 mRNA expression in C4-2 prostate cancer cells. Cells were treated with 50 μM of AhR inhibitor (CH223191) or 30 μM of Src inhibitor (PP2) alone or in combination or with vehicle control (DMSO) for 72 h and total RNAs were isolated and quantitative RT-PCR was performed to determine the mRNA expression of each target in treated cells. mRNA levels were normalized using L-19 which serves as an internal control. Each bar represents mean±SEM (n  =  3) and were analyzed by student t-test. (*) denotes statistically significant differences (*P<0.05) compared to control.

To confirm the synergistic effect of CH223191 and PP2 on AhR and AR activity, qRT-PCR was used to quantify mRNA expression of AhR responsive genes (AhR and CYP1B1) and AR responsive genes (AR and KLK3). Since AhR is constitutively active within the C4-2 cell line [15], CH223191 reduced AhR and CYP1B1 gene expression more than twofold when compared to the control. While PP2 alone also decreased AhR and CYP1B1 gene expression, inhibition of gene targets was further enhanced when the cells were treated with both CH223191 and PP2. CYP1B1 gene expression was found to be very similar to the expression of AhR when treated with CH223191 and PP2 as well as when both drugs were used in combination. Elevated AhR and CYP1B1 gene expression reported before tumor formation in a rat model of mammary tumorigenesis suggested differential CYP1B1 regulation by a constitutively active AhR. CYP1B1 expression was diminished by repression of AhR activity [18]. Because of the fundamental role of androgens in prostate development as well as prostate cancer, our objective was to investigate if the effect of CH223191/ PP2 also affected gene expression of AR and downstream target gene KLK3 (PSA). 50 μM CH223191 reduced AR gene expression by 60% in C4-2 cells. An identical decrease in AR expression was observed in response to treatment with PP2. These results helped to confirm that these drugs do not only decrease the expression of AhR but AR as well. Additionally, when C4-2 cells are co-treated with CH223191/PP2 AR expression is reduced by 85%. Furthermore, KLK3 which encodes for the glycoprotein prostate specific antigen (PSA) was observed to have a 65% and 70% decrease in expression in the presence of CH223191 and PP2 respectively. Yet, when these two drugs were used in combination the level of KLK3 mRNA expression was decreased even further to more than 97%. Therefore, it can be concluded that these drugs have an inhibitory effect on AhR and AR even more so when used in combination [Fig 3].

### Simultaneous reduction of AhR and Src signaling significantly reduces proliferation

**Fig 4:**
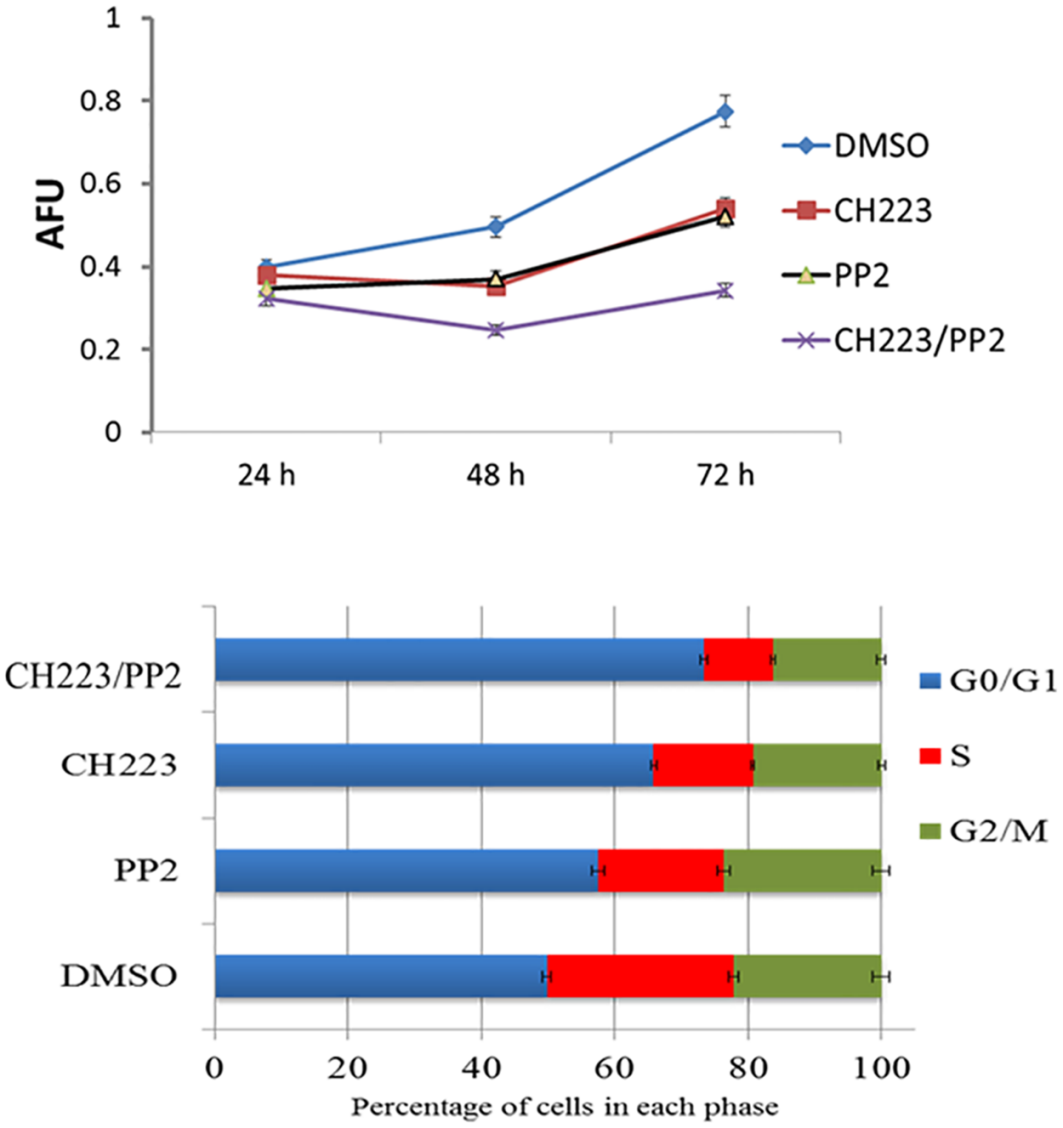
CH223191 and PP2 have a synergistic effect on proliferation of C4-2 prostate cancer cells. Cells were grown in 96 well plates at 5.0×10^3^ cells per well. The cells were treated with DMSO or 50 μM of CH223191 or 30 μM of PP2 alone or in combination for 24–72 hrs. (A) Cell growth was measured using Promega CellTiter 96 Cell Proliferation Assay per manufactures instructions. Each bar represents mean±SEM (n  =  3), *p <.05. (B) The cells were plated at a density of 1x10^6^ cells/dish and exposed to DMSO or 50 μM of CH223191 or 30 μM of PP2 alone or in combination for 72 hrs. Cells were harvested and analyzed for cell cycle. The cells with DMSO exposure served as control. Bar graphs represent mean ± SD of three separate experiments.

While expression and activity data confirmed the effectiveness of combination therapy on AR, next we examined those effects on the growth of C4-2 cells. Asim et al. showed incubation of C4-2 cells with Src inhibitor for six weeks led to a 50% decrease in cell growth [19]. The influence of AhR and Src on androgen independent cell line growth was observed when C4-2 cells were grown for 24–72 hours in the presence and absence of CH223191 and PP2. There was no significant difference between the four treatments on the rate of growth after 24 hours exposure. Growth of C4-2 cells was significantly inhibited in the presence of CH223191 and PP2 at 48 and 72 hours. Furthermore, CH223191/PP2 demonstrated a synergistic effect on growth inhibition at 48–72 hours. After 72 hours of exposure to the combination, C4-2 cells exhibited a 50% decrease in overall growth rate compared to DMSO and compared to 25% when CH223191 or PP2 were used alone. Together these finding show co-inhibition of AhR and Src synergistically reduce the growth rate of C4-2 PCa cells [Fig 4A]. Cell cycle analysis revealed an increase in the percent of cells in the G0/G1 phase of the cell cycle when cells were treated with either CH223191 or PP2. The percentage of cells remaining in G0/G1 was further increased when cells were co-treated with CH223191 and PP2 corresponding with the reduced growth rate seen with simultaneous inhibition of AhR and Src [Fig 4B].

## Discussion

Tyrosine kinase inhibitors (TKIs) have been extensively studied as a treatment for multiple malignancies [20]. Src is one of the TKIs shown to act as a regulator between intercellular and membrane proteins. Src activation, which has been reported in multiple types of cancers, can result in activation or repression of signaling pathways. [21,22,23]. Studies have also shown direct interaction between Src and AR. Src was shown to enhance the tumor progressive properties of AR in the absence of specific ligands to activate AR. In LNCaP prostate cancer cells, EGFR was shown to induce Src interaction with AR through the proline-rich 372–379 region within the SH3 domain. This interaction resulted in Src activation and enhanced cell cycle progression. Furthermore, a small peptide specifically designed to block AR and Src interaction increased apoptosis in LNCaP tumor xenografts [24]. These reported actions of Src make it an ideal target for treatment of men with advance stages of prostate cancer [25]. However, monotherapy designed clinical trials using Src inhibitors have had limited success and combination therapy may prove more beneficial.

Considering Src is an integral component of the cytosolic AhR complex, AhR activity may lead to persistent phosphorylation of AR by Src kinase even in the presence of Src inhibitors [26]. Coimmunoprecipitation experiments revealed that AhR forms a protein complex with Src and regulates activity by phosphorylating Src (Tyr416) and dephosphorylating Src (Tyr527) [27]. Immunoprecipitation assays revealed the association of AR with Src, suggesting complex formation among them [28]. Other studies have shown that Src kinase can cause AR transactivation in C4-2 PCa cells. In these studies, a specific Src kinase inhibitor resulted in decreased AR activation and invasion of C4-2 cells [19]. Previous evidence that Src can facilitate crosstalk between AhR and other transcription factors supports the premise that Src is capable of mediating crosstalk between AhR and AR. Src mediated crosstalk between AhR and epidermal growth factor receptor. The resulting crosstalk enhanced proliferation of colon cancer cells [27].

The molecular mechanism of AhR and AR crosstalk needs to be investigated further and could be multifaceted. Based of know mechanism of AhR interactions, the receptor may induce activation of AR by direct heterodimerization and overlapping coactivators as well as protein phosphorylation [15].

Our findings indicate that co-inhibition of Src and AhR represses AR function in a synergistic manner. Co-inhibition of AhR and Src with CH223191 and PP2 respectively inhibited AR phosphorylation and nuclear localization. This inhibition resulted in decreased AR transcriptional activity as evidenced by a significant decrease in the activity of an androgen responsive element luciferase assay and expression of androgen responsive genes. Consequently growth of CRPC cell line, C4-2, was significantly inhibited by co-targeting AhR and Src when compared to individual inhibition of both pathways. AR signaling is essential for the progression of prostate cancer. Simultaneous inhibition of AhR and Src could abolish AR signaling and decrease mortality associated with CRPC.
